# miR-143 Overexpression Impairs Growth of Human Colon Carcinoma Xenografts in Mice with Induction of Apoptosis and Inhibition of Proliferation

**DOI:** 10.1371/journal.pone.0023787

**Published:** 2011-08-25

**Authors:** Pedro M. Borralho, André E. S. Simões, Sofia E. Gomes, Raquel T. Lima, Tânia Carvalho, Duarte M. S. Ferreira, Maria H. Vasconcelos, Rui E. Castro, Cecília M. P. Rodrigues

**Affiliations:** 1 Research Institute for Medicines and Pharmaceutical Sciences - iMed.UL, Faculty of Pharmacy, University of Lisbon, Lisbon, Portugal; 2 Cancer Drug Resistance Group, Institute of Molecular Pathology and Immunology of the University of Porto (IPATIMUP), Porto, Portugal; 3 Centre of Medicinal Chemistry - University of Porto (CEQUIMED-UP), Porto, Portugal; 4 Instituto Gulbenkian de Ciência, Oeiras, Portugal; 5 Centro de Investigação em Patobiologia Molecular, Instituto Português de Oncologia de Francisco Gentil, Centro de Lisboa, Lisbon, Portugal; 6 Department of Biological Sciences, Faculty of Pharmacy, University of Porto, Porto, Portugal; 7 Department of Biochemistry and Human Biology, Faculty of Pharmacy, University of Lisbon, Lisbon, Portugal; Institut Jacques Monod, France

## Abstract

**Background:**

MicroRNAs (miRNAs) are aberrantly expressed in human cancer and involved in the (dys)regulation of cell survival, proliferation, differentiation and death. Specifically, miRNA-143 (miR-143) is down-regulated in human colon cancer. In the present study, we evaluated the role of miR-143 overexpression on the growth of human colon carcinoma cells xenografted in nude mice (immunodeficient mouse strain: N: NIH_(s)_ II-*nu/nu*).

**Methodology/Principal Findings:**

HCT116 cells with stable miR-143 overexpression (*Over-143*) and control (*Empty*) cells were subcutaneously injected into the flanks of nude mice, and tumor growth was evaluated over time. Tumors arose ∼ 14 days after tumor cell implantation, and the experiment was ended at 40 days after implantation. miR-143 was confirmed to be significantly overexpressed in *Over-143 versus Empty* xenografts, by TaqMan® Real-time PCR (*p*<0.05). Importantly, *Over-143* xenografts displayed slower tumor growth compared to *Empty* xenografts from 23 until 40 days *in vivo* (*p*<0.05), with final volumes of 928±338 and 2512±387 mm^3^, respectively. Evaluation of apoptotic proteins showed that *Over-143 versus Empty* xenografts displayed reduced Bcl-2 levels, and increased caspase-3 activation and PARP cleavage (*p*<0.05). In addition, the incidence of apoptotic tumor cells, assessed by TUNEL, was increased in *Over-143 versus Empty* xenografts (*p*<0.01). Finally, *Over-143 versus Empty* xenografts displayed significantly reduced NF-κB activation and ERK5 levels and activation (*p*<0.05), as well as reduced proliferative index, evaluated by Ki-67 immunohistochemistry (*p*<0.01).

**Conclusions:**

Our results suggest that reduced tumor volume in *Over-143 versus Empty* xenografts may result from increased apoptosis and decreased proliferation induced by miR-143. This reinforces the relevance of miR-143 in colon cancer, indicating an important role in the control of *in vivo* tumor progression, and suggesting that miR-143 may constitute a putative novel therapeutic tool for colon cancer treatment that warrants further investigation.

## Introduction

microRNAs (miRNAs or miRs) are endogenously encoded short non-coding RNAs (20–23 nt), pivotal players in posttranscriptional gene silencing of target mRNAs. In mammals, incomplete complementarity binding of the mature miRNA to the 3′UTR of target mRNA results in target gene silencing via translational repression, or in some cases via mRNA degradation [1].

The strong focus on miRNA research in recent years has lead to an exponential growth in the number of identified miRNAs, which exceed 1000 in humans [2] and putatively regulate over 60% of human genes [3]. Importantly, miRNAs are involved in the regulation or fine-tuning of a myriad of crucial biological processes commonly de-regulated in cancer, including cell proliferation, differentiation, cell-cycle and apoptosis, among others [4], [5]. Furthermore, it is now well known that miRNAs are aberrantly expressed in several forms of human cancer, including colon cancer [6], [7], [8]. However, and notwithstanding the fast growth of knowledge on miRNAs, only a small fraction of the molecular signaling circuitry regulated by miRNAs is known in cancer.

miR-143 expression has been reported as down-regulated in colon cancer, both in adenomas [9], [10] and colon carcinomas [6], [9], as well as in colon cancer cell lines [11], [12]. Further, miR-143 relevance as a putative cancer biomarker is growing, as it is down-regulated in various other human cancers, including B-cell malignancies [13], non-small cell lung cancer [14], esophageal squamous cell carcinoma [15], esophageal adenocarcinoma [16], osteosarcoma [17], bladder [18], cervical [19], prostate [20], and gastric [21] cancer. In addition, miR-143 is considered a pivotal regulator of gene expression, since it directly targets multiple mRNAs coding for proteins involved in cell proliferation, differentiation, survival and apoptosis, including KRAS [22], [23], DNMT3A [24], ELK1 [25], MYO6 [26], Bcl-2 [17] and ERK5 [27]. Interestingly, ERK5 is the most widely reported direct target of miR-143, which is downregulated by miR-143 overexpression [11], [12]. Finally, growing evidence supports an anti-proliferative, pro-apoptotic and chemosensitizer role for miR-143 in colon cancer, since it reduces cell viability and increases sensitivity to 5-fluorouracil (5-FU), the drug of choice in colorectal cancer treatment and a known inducer of apoptosis in colon cancer cell lines [28], [29].

Increased expression of mature miR-143 was found to occur following p53 up-regulation by doxorubicin in HCT116 colon cancer cells [22], and also in response to 5-FU exposure [12]. Furthermore, miR-143 may be involved in apoptosis proceeding via the intrinsic and/or extrinsic pathways, since it down-regulates anti-apoptotic protein Bcl-2, and is up-regulated after Fas-mediated apoptosis. The latest is accompanied by ERK5 downregulation [27], which we have previously demonstrated to directly induce apoptosis and chemosensitization in ERK5 siRNA-mediated knockdown experiments in colon cancer cells [12].

ERK5 is a mitogen-activated protein kinase (MAPK), activated by a wide range of cellular stresses and mitogens, and involved in the regulation of cellular survival, differentiation and proliferation. Importantly, ERK5 targets c-Myc, cyclin D1 and nuclear factor (NF)-κB, well known cell proliferation regulators [30]. In particular, NF-κB is involved in the promotion of cell proliferation and suppression of apoptosis, playing a pivotal role in tumor progression. NF-κB is constitutively activated in several malignant cells, including colon cancer [31], [32]. Importantly, ERK5 activation of NF-κB is involved in cellular transformation [33] and is critical for normal progression of the cell cycle from G2-M and timely mitotic entry [34]. Inhibition of NF-κB activation may be useful in antitumor therapy by increasing colon cancer cell sensitivity to 5-FU [35]. In addition, we have recently demonstrated that miR-143 overexpression significantly increases *in vitro* HCT116 colon cancer cell sensitivity to 5-FU, with a marked decrease in ERK5, NF-κB, and Bcl-2 steady-state levels [12]. This suggests that miR-143 overexpression in colon cancer cells may be an important strategy to reduce tumor growth and aggressiveness, and increase chemotherapy response.

In the present study, we evaluated the role of miR-143 overexpression on growth of HCT116 human colon carcinoma cells xenografted in mice, and the putative involvement of apoptosis and proliferation on miR-143 mechanism of action. Our results show that miR-143 markedly reduces human colon cancer cell xenograft growth *in vivo*, causing increased tumor cell apoptosis and decreased proliferation. In addition, miR-143 overexpression in human tumor xenografts in mice leads to significantly reduced NF-κB activation, and ERK5 expression and activation. These results underscore the relevance of miR-143 in colon cancer, suggesting an important role in the control of tumor progression *in vivo*, and expanding its anti-proliferative, pro-apoptotic and chemosensitizer role that we had previously demonstrated *in vitro*.

## Results

### miR-143 increases HCT116 cell growth inhibition

**Figure 1:**
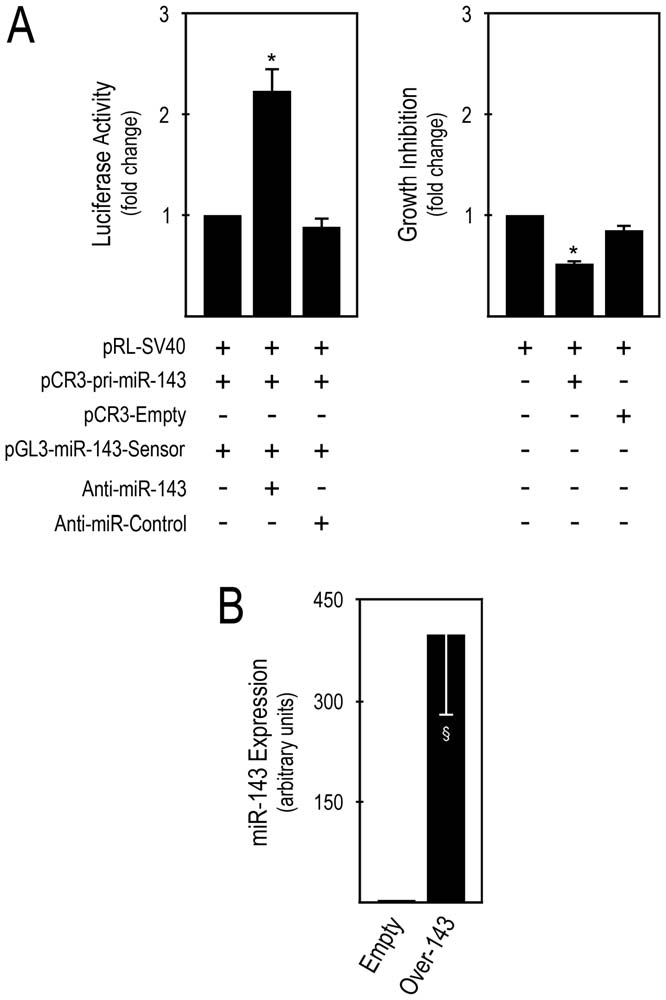
Mature miR-143 overexpression increases cell growth inhibition. A, HCT116 cells were co-transfected with the indicated plasmids and anti-miR inhibitors, and analyzed 48 h post-transfection. Cells were lysed and firefly and renilla luciferase activities determined by the dual luciferase assay (*left panel*). Cell growth inhibition was evaluated by MTS metabolism assays, and cells were then lysed for dual luciferase assay, to normalize the MTS metabolism assay (*right panel*). B, HCT116 cells were transfected with pCR3-pri-miR-143 and pCR3-empty and selected with G418, to generate *Over-143* and *Empty* cells, respectively. Cells were harvested for total RNA extraction after 5 to 25 days of selection. miR-143 expression was evaluated from 1.4 µl cDNA of 10 ng total RNA RT reactions, using specific primers for miR-143 and RNU6B for normalization. miR-143 expression levels were calculated by the ãã*C*_t_ method, using *Empty* cells as calibrator. Results are expressed as mean±SEM of at least 3 independent experiments. *p<0.05 from controls and §*p*<0.01 from *Empty*.

We initially confirmed the production of mature miR-143 from the pCR3-pri-miR-143 vector, and the effect of transient miR-143 overexpression from this DNA vector on HCT116 colon carcinoma cell growth. This was performed by co-transfection of pCR3-pri-miR-143 (miR-143 expression vector), a firefly luciferase miR-143 reporter for mature miR-143 detection (miR-143 sensor), and with either miR-143 specific inhibitor (anti-miR-143), or control (anti-miR-control). pRL-SV40 was also co-transfected and used as a normalization control. Our results clearly show that mature miR-143 was expressed from pCR3-pri-miR-143, since reduction of mature miR-143 bioavailability via anti-miR-143 co-transfection led to a significant increase in firefly activity (Figure 1A, left panel, middle bar), as compared to controls (*p*<0.05). Importantly, transient miR-143 overexpression significantly increased cell growth inhibition (Figure 1A, right panel, middle bar), compared to empty vector transfected cells (*p*<0.05), as evaluated by the MTS metabolism assay.

To evaluate the impact of miR-143 overexpression on the growth of human colon cancer cells xenografted in mice, we next produced HCT116 cells with stable miR-143 overexpression (*Over-143*) and control cell lines (*Empty*), by transfection with pCR3-pri-miR-143 or pCR3-empty, respectively, followed by selection and propagation of stably transfected cells with G418. *Over-143* and *Empty* cells with 5 to 25 days of G418 selection were processed for total RNA extraction to evaluate mature miR-143 expression by TaqMan® Real-time PCR using specific primers for mature miR-143, and RNU6B for normalization to endogenous control. Importantly, our results show that high and stable mature miR-143 expression was consistently obtained in *Over-143*, as compared to *Empty* cells (Figure 1B) (*p*<0.01).

### miR-143 overexpression decreases the growth of HCT116 human colon carcinoma cells xenografted in mice

**Figure 2:**
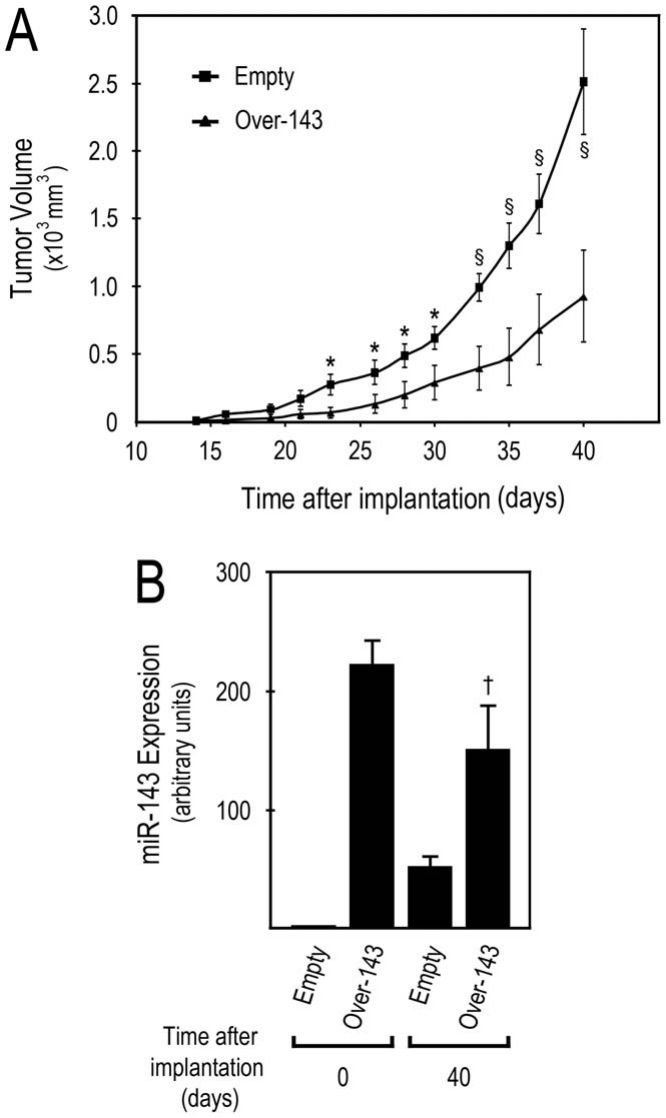
miR-143 overexpression decreases human colon carcinoma cell tumor xenograft growth in nude mice. Forty-eight hours after release from dual-thymidine block, 5×10^5^ miR-143 overexpressing (*Over-143*), or control (*Empty*) cells, were s.c. injected into the flanks of nude mice. A, Tumor xenograft size was regularly measured (every 2-3 days), from the initial signs of tumor development, until the end of the experiment (∼ 14 and 40 days after cell implantation, respectively), to plot tumor growth curves. Animals were then sacrificed and tumors excised. B, Total RNA was extracted from *Over-143* and *Empty* cells prior to implantation into animals (t = 0 days) and from snap frozen tumor xenograft samples (t = 40 days), and used to quantify mature miR-143 expression. miR-143 expression was evaluated from 1.4 µl cDNA of 10 ng total RNA RT reactions, using specific primers for miR-143 and RNU6B for normalization. miR-143 expression levels were calculated by the ãã*C*_t_ method, using *Empty* cells as calibrator. Two independent experiments were performed using a total of 12 animals for *Over-*143 and 10 animals for *Empty*. Results are expressed as mean±SEM. *p<0.05, §*p*<0.01 from *Over-143* and †*p*<0.05 from *Empty*.

Having validated our cell model *in vitro*, we next evaluated the *in vivo* effect of miR-143 overexpression on HCT116 tumor xenograft growth. *Over-143* or *Empty* cell suspensions were injected s.c. into the flanks of 6-week-old immunodeficient mice, and tumor growth was evaluated and registered periodically, to plot tumor growth curves. Tumors arose ∼ 14 days after subcutaneous administration, and tumorigenicity studies were terminated at day 40 after cell implantation. Our results demonstrate a significant and marked reduction of tumor volume in *Over-143* xenografts compared to *Empty* xenografts, from day 23 after implantation until the end of the experiment, at day 40 (Figure 2A) (*p*<0.05 and *p*<0.01 from 23 to 30, and from 33 to 40 days after implantation, respectively). Therefore, *Over-143* xenografts showed a significantly slower tumor growth compared to the *Empty* xenografts, displaying final tumor volumes of 928±338 and 2512±387 mm^3^, respectively.

Tumors were collected after sacrificing the animals at the end of *in vivo* tumor growth evaluation, 40 days after *Over-143* or *Empty* cell implantation. Subsequently, total RNA was extracted from *Over-143* and *Empty* xenografts (t = 40), and also from *Over-143* and *Empty* cells before injection into nude mice (t = 0). RNAs were then used to evaluate mature miR-143 expression. Our results show that at tumor cell implantation (t = 0), miR-143 levels were more than 200-fold increased in *Over-143* compared to *Empty* cells, demonstrating that the injected cell lines were in the desired experimental conditions at implantation (Figure 2B). Importantly, after 40 days of xenograft growth *in vivo*, *Over-143* xenografts still presented a significant increase in mature miR-143 expression compared to *Empty* xenografts (*p*<0.05) (Figure 2B).

### miR-143 overexpressing tumor xenografts display reduced ERK5 steady-state levels, and NF-κB nuclear translocation

**Figure 3:**
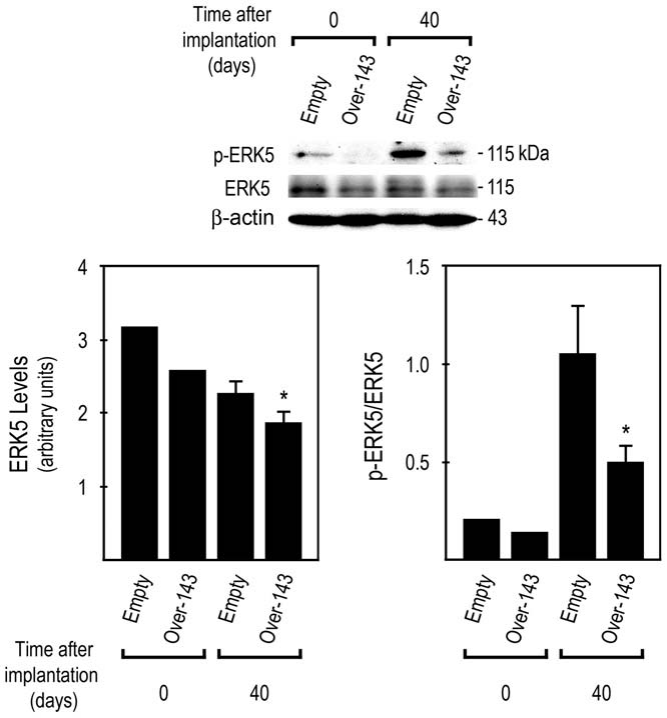
miR-143 overexpression decreases ERK5 expression and activation in tumor xenografts. Total proteins were extracted from *Over-143* and *Empty* cells prior to implantation into animals (t = 0 days) and from snap frozen tumor xenograft samples (t = 40 days), and used to evaluate ERK5 and p-ERK5 steady-state levels by Western blot. Results are expressed as mean±SEM. *p<0.05 from *Empty*.

miR-143 directly regulates the expression of several proteins involved in crucial biological processes, whose deregulation is commonly associated with cancer. Importantly, transient overexpression of miR-143 mimetics in HCT116 [12], SW480 and DLD-1 [11] colon cancer cell lines has been shown to down-regulate ERK5 steady-state levels. In addition, modulating mature miR-143 levels by transient co-tranfection of miR-143 mimetics and inhibitors regulates ERK5 protein [12]. Furthermore, activated ERK5 modulates cell survival, differentiation and proliferation, through c-Myc, cyclin D1 and NF-κB activation [30]. Therefore, we extracted total proteins from *Over-143* and *Empty* xenografts (t = 40) and from *Over-143* and *Empty* cells prior to implantation into nude mice (t = 0), and evaluated ERK5 steady-state levels by Western blot (Figure 3, top). Our results demonstrate that *Over-143* xenografts displayed reduced ERK5 protein levels compared to *Empty* xenografts (Figure 3, bottom left) (*p*<0.05). Furthermore, we also evaluated p-ERK5, the dual phosphorylated (Thr218/Tyr220) active form of ERK5. *Over-143* xenografts presented reduced p-ERK5 compared to *Empty* xenografts, which resulted in decreased ratios of p-ERK5/total ERK5 (Figure 3, bottom right) (*p*<0.05).

**Figure 4:**
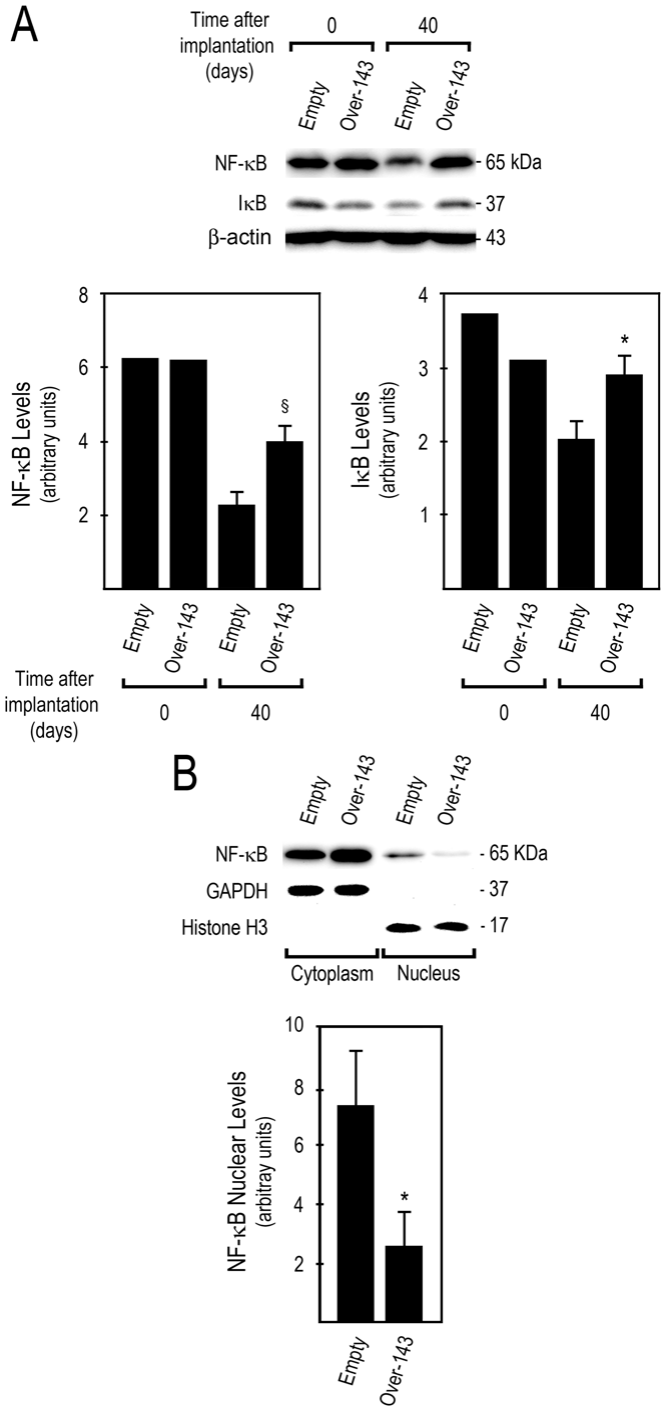
miR-143 overexpression decreases NF-κB nuclear translocation in tumor xenografts. A, Total proteins were extracted from *Over-143* and *Empty* cells prior to implantation into animals (t = 0 days) and from snap frozen tumor xenograft samples (t = 40 days), and used to evaluate NF-κB and IκB steady-state levels by Western blot. B, cytosolic and nuclear proteins were extracted from snap frozen tumor xenograft samples (t = 40 days; *Over-143* and *Empty*), to evaluate NF-κB nuclear translocation by Western blot. Results are expressed as mean±SEM. *p<0.05 and §*p*<0.01 from *Empty*.

Evidence suggests that ERK5 activation of NF-κB promotes cellular transformation [33], and that NF-κB is a critical factor for G2–M cell cycle progression and timely mitotic entry [34]. NF-κB is also involved in inhibition of apoptosis and stimulation of cellular growth, thus contributing to tumor promotion, and chemoresistance. Curiously, our results indicate that NF-κB steady-state levels were increased in *Over-143* xenografts at day 40, compared to *Empty* xenografts (Figure 4A, bottom left) (*p*<0.01). Nevertheless increased steady-state levels of IκB in *Over-143 versus Empty* xenografts at day 40 (Figure 4A, bottom right) (*p*<0.05) suggest that NF-κB is less activated in *Over-143* xenografts. This was further confirmed by the reduced ratio between nuclear and cytoplasmic NF-κB levels in *Over-143* compared to *Empty* xenografts (Figure 4B) (*p*<0.05).

### miR-143 overexpressing tumor xenografts display increased apoptosis and reduced proliferation

**Figure 5:**
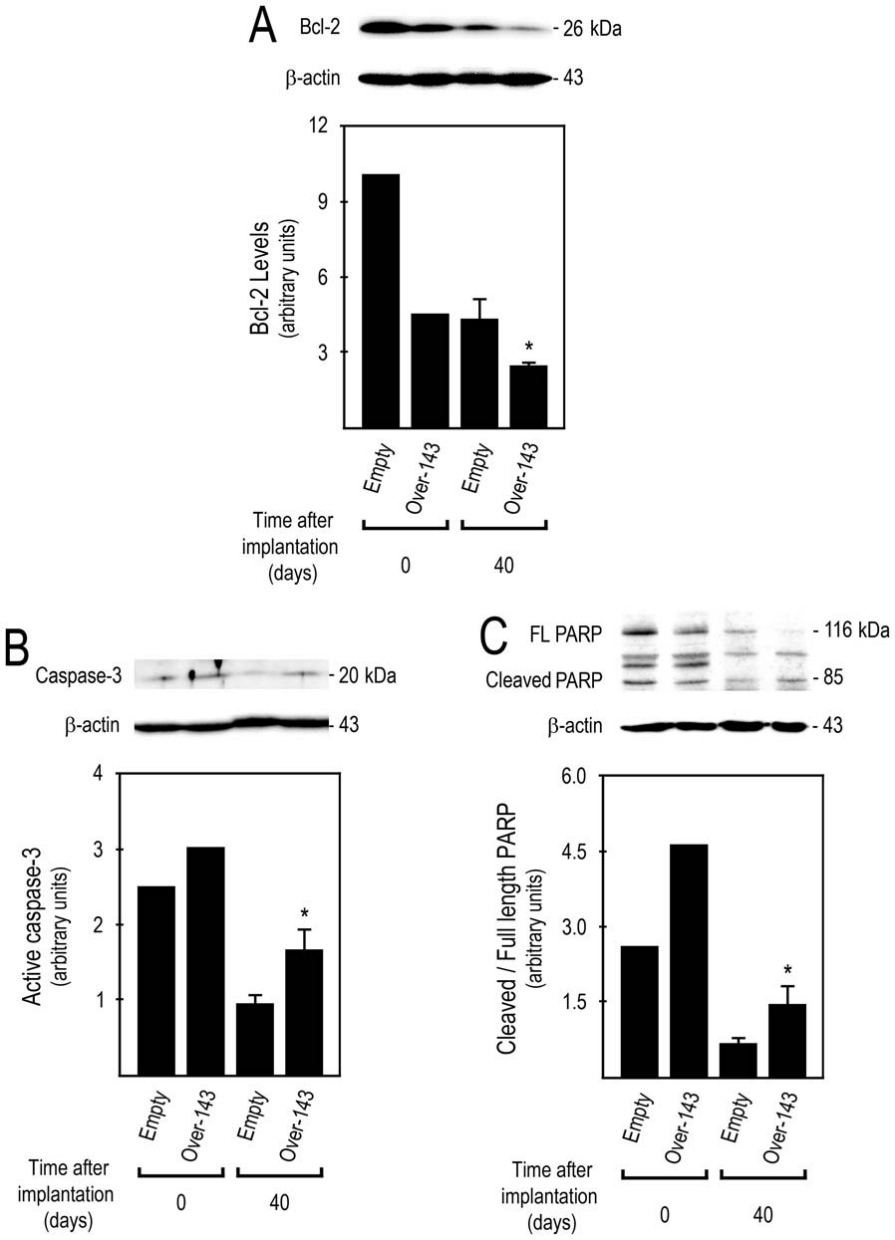
miR-143 overexpression reduces Bcl-2, and increases caspase-3 processing and PARP cleavage in tumor xenografts. Total proteins were extracted from *Over-143* and *Empty* cells prior to implantation into animals (t = 0 days) and from snap frozen tumor xenograft samples (t = 40 days), and used to determine by Western blot: A, steady state levels of Bcl-2; B, active caspase-3; and C, PARP cleavage. Results are expressed as mean±SEM. FL, full length. *p<0.05 from *Empty*.

**Figure 6:**
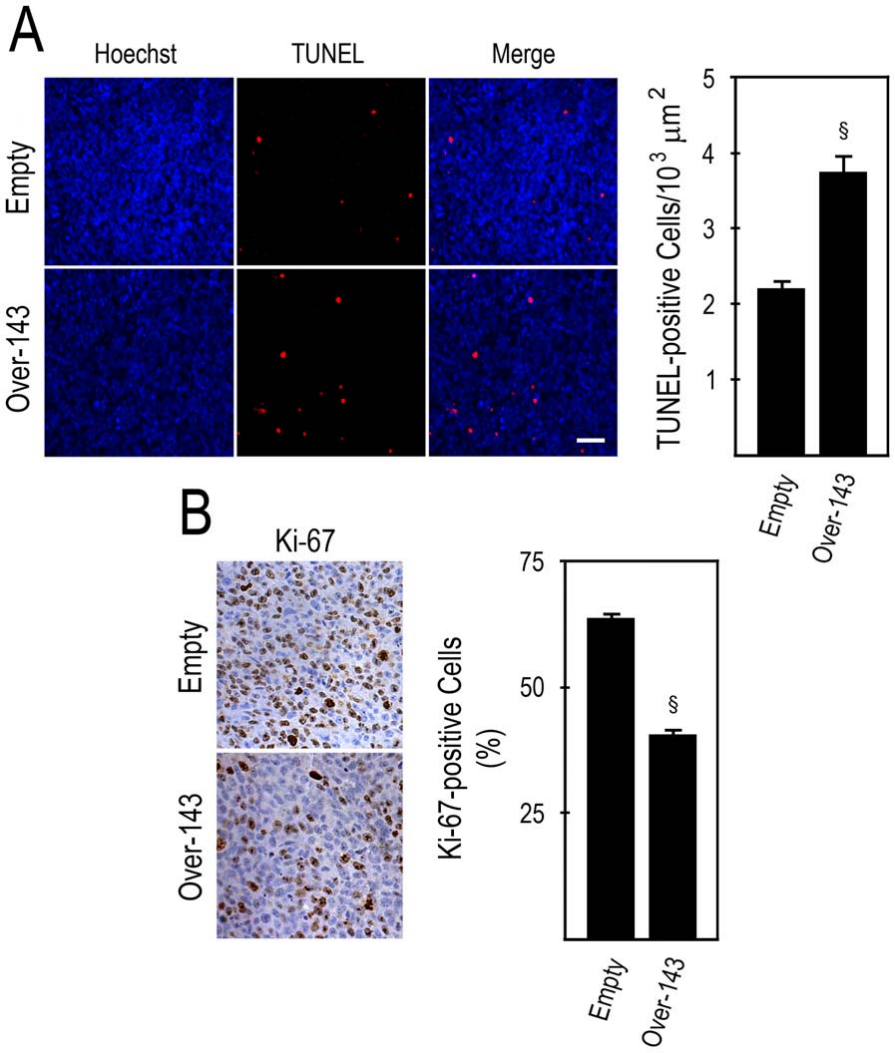
miR-143 overexpression increases apoptosis and decreases proliferation in tumor xenografts. Tumor xenografts obtained by s.c. injection of *Over-143* or *Empty* cells were formalin fixed and paraffin embedded. Four and 3-µm-thick sections were cut and used for TUNEL assay and Ki-67 immunohistochemistry, respectively. A, Analysis of *in situ* detection of apoptotic cells in tumor sections of *Over-143* and *Empty* xenografts. Representative images of TUNEL assay (100x) (left panel), and number of TUNEL-positive cells (right panel) of *Over-143* and *Empty* xenografts. B, Analysis of cell proliferation by Ki-67 immunostaining in tumor sections of *Over-143* and *Empty* xenografts. Representative images of Ki-67 immunostaining (400x) (left panel), and percentage of Ki-67-positive cells (right panel) of *Over-143* and *Empty* xenografts. Results are expressed as mean±SEM. §*p*<0.01 from *Empty*.

Our results demonstrate that miR-143 overexpression increased colon tumor cell growth inhibition *in vitro*, and decreased the growth of tumor cells xenografted in mice. In addition, miR-143 overexpresion reduced ERK5 steady-state levels, ERK5 activation, and NF-κB nuclear translocation, suggesting regulation of colon cancer cell survival, and proliferation capabilities. Importantly, we and others have previously reported that ERK5 knockdown by RNA interference induces apoptosis *in vitro* [12], [36]. We next evaluated the effect of miR-143 on apoptosis and proliferation of human tumor xenografts in mice. *Over-143* xenografts displayed reduced steady-state levels of the anti-apoptotic protein Bcl-2 (Figure. 5A) (*p*<0.05), together with increased caspase-3 processing and PARP cleavage (Figure. 5B and C) (*p*<0.05). As expected, the frequency of TUNEL-positive cells was increased in *Over-143 versus Empty* xenograft tumoral tissue, which displayed 3.8±0.2 and 2.2±0.1 cells per 10^3^ µm^2^ of tumor area, respectively (Figure. 6A) (*p*<0.01). This data re-enforce the notion that induction of apoptosis is part of the *in vivo* mechanism of action of miR-143 in this xenografted tumor model. Importantly, immunohistochemistry for Ki-67 in xenograft tissue sections showed that the fraction of proliferating cells is lower in *Over-143 versus Empty* xenografts, with percentages of proliferation of 40.6± 1.1 and 63.7±0.9%, respectively (Figure. 6B) (*p*<0.01). Thus, miR-143 overexpression induces apoptosis and reduces proliferation of human colon carcinoma cells xenografted in mice, further re-enforcing the potential deleterious effects arising from loss of miR-143 expression in human colon cancer.

## Discussion

The relevance of miRNAs in human cancer is growing exponentially, with this class of small non-coding RNAs considered pivotal gene expression regulators, and carrying enormous potential as diagnostic, prognostic and therapeutic tools. Aberrant miRNA expression may negatively impact on the regulation of multiple important cellular processes, such as cell proliferation, differentiation and turnover, commonly de-regulated events in cancer. In particular, miR-143 is gaining increasing relevance as putative cancer biomarker and therapeutic tool in human cancer. Its reduced expression has been demonstrated in multiple cancer types, including those of the gastrointestinal tract. In addition to colon cancer, miR-143 has been reported down-regulated in oesophageal squamous cell carcinoma [15], oesophageal adenocarcinoma [16], and gastric cancer [21]. Further, we and others have previously demonstrated that miR-143 expression is reduced in human colon cancer [6], [7], [10], and also that miR-143 overexpression markedly reduces the viability of several colon cancer cell lines *in vitro* [11], [12]. In the present study, we evaluated the effect of miR-143 overexpression on HCT116 human colon cancer tumor xenograft growth in nude mice.

Initially, we demonstrated transient miR-143 overexpression from pCR3-pri-miR-143 in HCT116 cells, which significantly increased cell growth inhibition as compared to pCR3-empty transfection, in accordance with our previous results [12]. To evaluate the *in vivo* effect of miR-143 overexpression on colon cancer tumor xenograft growth, we next created HCT116-derived stable miR-143 overexpressing cells, and confirmed in at least three independent batches evaluated at multiple random selection times that miR-143 expression was consistently highly increased in *Over-143 versus Empty* cells, attesting to the robustness of miR-143 overexpression in our cell model. Such a high differential expression has not been reported before for miR-143 in human colon cancer samples. However, a recent report in ulcerative colitis patients, which are at increased risk for development of colorectal cancer, demonstrated that miR-143 expression is downregulated up to 20-fold, compared to normal colon [37]. Several other reports have shown differential miRNA expression of > 100-fold in biological samples using different miRNA detection methods and models [38], [39], [40], [41], [42], [43]. Importantly, we have also shown that miR-143 expression is 100-fold lower in SW480 as compared to HCT116 cells [12]. In addition, higher levels of miRNA overexpression can be achieved, without a corresponding effect on cell viability, or other cellular functions [44].

Stable miR-143 overexpression significantly decreased the rate of *xenografted* tumor growth, as clearly evidenced by the reduced *Over-143* tumor xenograft volumes as compared to *Empty*. Further, tumor xenografts from *Over-143* and *Empty* cells were first detectable 14 days after cell implantation. In the following week, the impairment of tumor growth in result of miR-143 overexpression was already evident, becoming statistically significant 9 days later, at 23 days after cell implantation. Our results clearly demonstrate that miR-143 overexpression in colon cancer cells, delays xenografted tumor growth, highlighting the relevance of miR-143 as a putative therapeutic agent for the treatment of this disease. Interestingly, this is in agreement with a recent study using prostate cancer cell lines, in which repeated xenograft intratumoral injection (3x) of miR-143 mimetics at 5000 nM, followed by *in vivo* electroporation, resulted in tumor growth abrogation or decrease, in mice grafted with LNCaP and C4-2 prostate cancer cells, respectively [20]. Mature miR-143 expression was increased from 1 to 3 fold in LNCaP and C4-2 xenografts. Here, we demonstrate that we are able to maintain higher levels of mature miR-143 expression throughout the xenografted tumor growth evaluation period, by transfecting HCT116 cells *in vitro* and selecting with G418 prior to subcutaneous injection, which resulted in a marked decrease in colon cancer xenograft tumor growth. Our results are also in line with another recent study where liposome entrapped 3′-modified miR-143 mimetics were administered by intravenous injection following a regimen of weekly administration (5x), in mice xenografted with human DLD-1 colon cancer tumors [9]. The authors reported a higher stability of the miR-143 mimetics to nuclease degradation and a significant dose-dependent decrease in tumor xenograft size in modified-miR-143 treated mice as compared to control miRNA (non-specific sequence), with a final tumor xenograft size reduction of ∼ 50% two weeks after the five week treatment at a dose of 50 µg per mouse.

ERK5 is the most widely reported miR-143 direct target in colon cancer, and ERK5 signaling is involved in the regulation of cell survival, differentiation, proliferation and apoptosis. Our results demonstrated a significant reduction in ERK5 and p-ERK5 in *Over-143 versus Empty* xenografts. This suggests that in human colon cancer cell tumor xenografts, ERK5 is targeted by miR-143, leading to reduced protein steady-state levels and activation. These results further expand previous reports of miR-143-mediated ERK5 expression knockdown in colon cancer cell lines *in vitro* [11], [12], and are in agreement with reduced ERK5 expression following repeated (3x) miR-143 intratumoral injection and *in vivo* electroporation in prostate cancer cell tumor xenografts [20]. In addition, ERK5 activates multiple cellular proteins involved in the regulation of cell proliferation and survival, including NF-κB. ERK5 activation of NF-κB appears to be an extremely relevant biological event, since it has been implicated in cellular transformation [33], cell cycle progression from G2 to mitosis, and timely mitotic entry [34]. Curiously, our results show that *Over-143* xenografts displayed increased expression of NF-κB, compared to *Empty* xenografts. However, we also found increased expression of IκB in *Over-143 versus Empty* xenografts, suggesting that NF-κB may not be increasingly activated. In fact, reduced NF-κB nuclear translocation in *Over-143 versus Empty* xenografts confirmed a significant reduction of NF-κB activation. This suggests that miR-143 overexpression diminishes ERK5 expression and activation, which in turn may lead to reduced NF-κB activation, thus reducing tumor cell proliferation and growth in this *in vivo* tumor model. This is also supported by previous studies, where shRNA-mediated ERK5 knockdown in T lymphoma cell line EL-4 decreased nuclear accumulation of the NF-κB p65 subunit, while ERK5 activation led to constitutive nuclear localization of p65 and increased activation [45]. Further, stimulation of ERK5 was shown to activate NF-κB via ribosomal S6 kinase 2 (RSK2)-mediated phosphorylation, and degradation of IκB [34]. In addition, nuclear translocation of NF-κB is greater in proliferative compared to resting phase colon cancer cells [31]. Importantly, ERK5 knockdown abrogated the growth of EL-4 subcutaneous tumors in mice [45]. In addition, the ERK5/NF-κB axis may be an important signaling pathway in mediating sensitivity to anti-cancer drugs. Similar to the effects we reported after exposure of HCT116 colon cancer cells overexpressing miR-143 to 5-fluorouracil [12], exposure of MDA-MB-231 breast cancer cells to genistein induced cell growth suppression and induction of apoptosis, with down-regulation of ERK5, p-ERK5, NF-κB and Bcl-2 steady state levels. Interestingly, genistein exposure markedly reduced NF-κB DNA binding activity, via MEK5/ERK5 pathway inhibition [46], which raises the possibility that miR-143 may also be involved in genistein mechanism of cytotoxicity. Collectively, these data underscore the relevance of ERK5/NF-κB signaling for xenografted tumor proliferation and growth, and highlight the pivotal role of miR-143 in the regulation of this molecular signaling pathway. The early loss of miR-143 expression in the transition of normal colon to adenoma [9], may be a key event in colon cancer tumorigenesis by allowing unchecked cell proliferation, and this may also contribute to tumor growth and progression.

Another important aspect of miR-143 allowing the control of tumor growth may be its putative pro-apoptotic role. In this regard, it has been demonstrated that miR-143 overexpression significantly decreases the steady-state levels of anti-apoptotic protein Bcl-2 [12], [17], inducing apoptosis and sensitization to Fas-induced apoptosis [27]. In addition, we have also shown that miR-143 induces sensitization of HCT116 colon cancer cell line to 5-fluorouracil-induced apoptosis [12], which in turn is Fas-dependent in this cell type [28]. Here, we show that *Over-143 versus Empty* xenografts displayed decreased steady-state levels of Bcl-2, and increased caspase-3 activation and PARP processing, suggesting that miR-143 overexpressing xenografts may present higher levels of tumor cell apoptosis.

Finally, to confirm the indications that miR-143 may reduce tumor xenograft growth by reducing proliferation and increasing apoptosis of colon cancer cells, we evaluated apoptosis and proliferation in tumor xenograft tissue sections, by TUNEL assay and Ki-67 immunohistochemistry, respectively. Importantly, miR-143 overexpression significantly increased apoptosis, and decreased proliferation, which is consistent with the marked reduction in tumor growth. To our knowledge, this is the first demonstration that miR-143 overexpression induces apoptosis in human colon tumor cells xenografted in mice, and are in agreement with reported decreased proliferation ratio of LNCaP and C4-2 prostate cancer xenograft tumors injected with miR-143 and electroporated *in vivo* [20].

The results presented herein provide additional knowledge on miR-143 mechanism of action in human colon tumor cells xenografted in mice. miR-143 overexpression resulted in reduced tumor xenograft growth, with tumors presenting decreased proliferation and increased apoptosis. The mechanism of miR-143 action in this model is suggested to involve modulation of ERK5/NF-κB signaling pathways. Collectively, our data re-enforces the notion that miR-143 loss may be a pivotal event in colon cancer, suggesting an important function for miR-143 in the control of tumor progression *in vivo*. Additional studies are needed to further explore the re-introduction of miR-143 in colon cancer cells, as this may prove to be a valid a therapeutic approach for colon cancer treatment.

## Materials and Methods

### Ethics statement

All experimental procedures were carried strictly within the rules of the Portuguese official Veterinary Directorate (Direcção Geral de Veterinária, DGV), which follows the FELASA (Federation of European Laboratory Animal Science Associations) guidelines and recommendations concerning laboratory animal welfare. In this regard, animal experiments at IPATIMUP, University of Porto complied with the European Union legislations governing animal experimentation, namely the Convention for the Protection of Vertebrate Animals used for Experimental and other Scientific Purposes (ETS123), the EC recommendation n° 2007/526/CE and the Directive for the Protection of Vertebrate Animals used for Experimental and other Scientific Purposes (86/609/EEC), which was transposed to the National Laws through Decreto-Lei n° 129/92. Experiments further complied with the remaining national legislation for animal protection and welfare, namely Portaria n° 1005/92, Portaria n° 466/95, Decreto-Lei n° 197/96, and Portaria n° 1131/97.

### Cell culture

HCT116 human colorectal cancer cells [47] were grown in Dulbecco's modified Eagle's medium (DMEM) supplemented with 10% fetal bovine serum (Invitrogen Corporation, Grand Island, NY) and 1% antibiotic/antimycotic solution (Sigma-Aldrich, St. Louis, MO), and maintained at 37°C in a humidified atmosphere of 5% CO_2_.

### Transfection of miR-143 vectors and anti-miR-143 inhibitor

HCT116 cells were transiently transfected with miR-143 overexpression vector, coding for the miR-143 precursor (pCR3-pri-miR-143), and miR-143 sensor, comprising two sequences complementary to mature miR-143 sequence (pGL3-miR-143 sensor) [48]. pRL-SV40 (Promega, Madison, WI) was used for transfection normalization. pGL3-control plasmid (Promega) and pCR3-empty vector were used as negative controls. To further validate the experimental model, cells were co-transfected with anti-miR inhibitors, by adding 50 nM anti-miR-143 or anti-miR-control inhibitors (Applied Biosystems, Foster City, CA) to the vector mixture described above. Transfections were performed using lipofectamine 2000 (Invitrogen), according to the manufacturer's instructions.

### Evaluation of cell growth inhibition

Cell growth inhibition was evaluated with CellTiter96® AQueous Non-Radioactive Cell Proliferation Assay (Promega), using 3-(4,5-dimethylthiazol-2-yl)-5-(3-carboxymethoxyphenyl)-2-(4-sulfophenyl)-2H-tetrazolium inner salt (MTS), according to the manufacturer's instructions. Finally, cells were processed for luciferase assay and transfection efficiency normalization.

### Luciferase activity

Firefly and renilla luciferase activities were measured using the Dual-Luciferase® Reporter Assay System (Promega). Renilla luciferase activity was used as a transfection normalization control.

### Generation of HCT116 cells with stable expression of miR-143

miR-143 overexpression and control cell lines were prepared from HCT116 cells as previously described [12]. Briefly, ∼ 80% confluent HCT116 cells were trypsinized, counted and plated on 6 well plates at a density of 1.5×10^5^ cells/well. Twenty-four hours after plating, cells were transfected with 4 µg of miR-143 expression vector (pCR3-pri-mir-143) [48] or 4 µg of the respective empty vector control (pCR3-empty), using lipofectamine 2000 (Invitrogen), according to the manufacturer's instructions, to originate *Over-143* and *Empty* cell lines, respectively. Twenty four hours later, selection of transfected cells with 1 mg/ml G418 (Invitrogen) was initiated, with transfected cell populations being propagated and maintained by splitting sub-confluent cells into fresh complete media supplemented with 1 mg/ml G418, every 3 days. After three weeks of selection, miR-143 expression was evaluated by TaqMan® Real-time RT-PCR; after confirming miR-143 overexpression in *Over-143*, cells were xenografted in mice for evaluation of tumor growth in an *in vivo* model. In our previous *in vitro* studies, we have used a single clone with miR-143 overexpression in parallel with a miR-143 overexpressing cell population [12], similar to the one used in the present study. All reported effects were similar in both overexpression cell lines as compared to controls, with no marked changes in the effects from the miR-143 overexpression single clone cell line to the miR-143 overexpression cell population.

### Processing of cells for xenograft tumor growth evaluation

Prior to animal injection, cells were synchronized by dual-thymidine block as previously described [12]. Briefly, sub-confluent cell populations were trypsinized, counted, and plated on T150 flasks, at a density of 1.5×10^5^ cell/ml, using 20 ml complete media supplemented with 1 mg/ml G418. Eight hours after plating, 2 mM thymidine (Sigma-Aldrich) in ddH_2_0 was added to the culture media, and cells were cultured for 14 h. Cells were then released from first thymidine block by removing culture media, washing 3 times with PBS and by adding fresh complete media supplemented with 1 mg/ml G418, without thymidine. Ten hours later, cells were submitted to a second thymidine block, by replacing culture media with complete media supplemented with 1 mg/ml G418 and 2 mM thymidine. Fourteen hours later, cells were released from second thymidine block by removing culture media, washing 3 times with PBS and by adding fresh complete media supplemented with 1 mg/ml G418, without thymidine. Cells were grown for additional 48 h, prior to animal injection.

### Xenograft growth evaluation

Forty-eight hours after release from second block, cells were washed 3 times with PBS, trypsinized, counted and ressupended in DMEM at a density of 5×10^6^ cells/ml DMEM for animal injection. A total of 5×10^5^ *Over-143* or *Empty* cell line suspension (100 µl cell suspension), was injected subcutaneously into the flanks of 6-week-old immunodeficient nude mice (strain N:NIH(S)II-nu/nu). Two independent experiments were performed, using a total of 12 animals for *Over*-143 and 10 animals for *Empty* tumor xenograft growth evaluation. From the moment of injection, animals were inspected regularly for signs of tumor development, and the size of the tumors regularly measured with calipers every 2-3 days, recorded in an individualized chart, and used to calculate tumor volumes (WxLxH). Tumorigenicity studies in mice with subcutaneous tumors were terminated at day 40 after subcutaneous injection of cells, when mice were sacrificed. At excision, tumors were sectioned into two equal portions; one half was fixed overnight in 10% buffered formalin for subsequent paraffin embedding and sectioning; the other half was rinsed with sterile saline, snap frozen in liquid nitrogen and stored at -80°C for subsequent RNA and protein extraction.

### Total RNA extraction

Tissue from tumor xenografts was used for RNA extraction, together with cell pellets from *Over-143* and *Empty* cells prior to injection into nude mice (t = 0). Total RNA containing small RNA species was extracted from tissues and cell lines with TRIZOL® reagent (Invitrogen) according to the manufacturer's instructions. Samples were homogenized in TRIZOL® reagent using a motor-driven Bio-vortexer (No1083; Biospec Products, Bartlesfield, OK) and disposable RNAse/DNAse free sterile pestles (Thermo Fisher Scientific, Inc., Chicago, IL). RNA was quantified using a NanoDrop® spectrophotometer, and typically showed A260/280 ratios between 1.9 and 2.1.

### Mature miR-143 expression

Real-time PCR was performed to determine the expression level of mature miR-143, as previously described [12]. RT reactions were performed using 20 ng of total RNA extracted from tumor xenografts and also from cell lines prior to injection into the nude mice (t = 0), using a TaqMan® MicroRNA reverse transcription kit and TaqMan® MicroRNA assays specific for the mature form of hsa-miR-143, and human RNU6B for normalization to endogenous control. Real-time PCR reactions were performed using standard TaqMan® PCR reagents and TaqMan® MicroRNA assays for hsa-miR-143, and human RNU6B (all from Applied Biosystems). Triplicate reactions were run per sample. Data were collected with 7000 System Sequence Detection Software, version 1.2.3 (Applied Biosystems). The comparative threshold cycle method was used to calculate the amplification factor, where the threshold cycle (*C_t_*) is defined as the cycle number at which the fluorescence passes the fixed threshold intensity level. miR-143 expression levels in different samples were calculated on the basis of ΔΔC*_t_* method. *Empty* cells prior to injection into nude mice were used as the calibrator. The n-fold change in miR-143 expression was obtained using the formula: 2^-ΔΔC*t*^.

### Total, nuclear and cytosolic protein isolation

Total protein extracts were prepared from tumor xenograft tissues and cell lines. Samples were homogenized in ice-cold buffer containing 10 mM Tris-HCl, pH 7.6, 5 mM MgCl_2_, 1.5 mM KAc, 2 mM dithiothreitol (DTT) and protease inhibitor cocktail tablet (Roche Diagnostics GmbH, Mannheim, Germany), for 15 sec/tube using a motor-driven Bio-vortexer and disposable sterile pestles. Subsequently, an equal volume of ice-cold 2X total protein buffer, containing 10 mM Tris-HCl pH 7.6, 1% Nonidet-P40 and protease inhibitor cocktail tablet was added, and the samples were homogeneized by vortexing and incubated on ice for 30 min. Next, samples were sonicated for 30 sec and centrifuged at 10,000 g for 10 min, with total proteins being recovered in the supernatants. Total protein extracts were snap frozen in liquid nitrogen and stored at -80°C.

To evaluate nuclear translocation of NF-κB, cytoplasmic and nuclear extracts were prepared from tumor xenograft tissues. Samples were homogenized as described above for total protein extraction, and next centrifuged at 500 *g* for 10 min at 4°C. Cytosolic proteins were recovered in the supernatant. Nuclear pellets were washed in buffer containing 10 mM Tris-HCl, pH 7.6, 5 mM MgCl2, 0.25 M sucrose, 0.5% Triton X-100, and protease inhibitors, and then centrifuged at 500 *g*, resuspended, and sonicated in buffer containing 10mM Tris-HCl, pH 7.6, and 0.25 M sucrose with protease inhibitors. Finally, the suspension was centrifuged through 0.88 M sucrose at 2000 *g* for 20 min at 4°C, and nuclear proteins recovered in the supernatant.

### Immunoblotting

Steady-state levels of Bcl-2, caspase-3, PARP, ERK5, p-ERK5, NF-κB (p65), IκB, GAPDH and histone H3 proteins were determined by immunoblot analysis. Briefly, 50-75 µg of total protein extracts were separated on 6, 8 or 12% SDS-polyacrylamide electrophoresis gels. After electrophoretic transfer onto nitrocellulose membranes, immunoblots were incubated with 15% H_2_O_2_ for 15 min at room temperature. After blocking with 5% milk solution, the blots were incubated overnight at 4°C with primary mouse monoclonal antibody reactive to Bcl-2 (#sc-7382) and GAPDH (#sc-32233), or with primary rabbit polyclonal antibody reactive to caspase-3 (#sc-7148), PARP (#sc-7150), NF-κB (p65) (sc-372), or to IκB (sc-371) (all from Santa Cruz Biotechnology, Inc., Santa Cruz, CA). Blots were also incubated overnight at 4°C with primary rabbit polyclonal antibody reactive to ERK5, p-ERK5 (#3372 and #3371; Cell Signaling, Beverly, MA) or to histone H3 (#06-755; Millipore, Billerica, MA). Finally, the immunoblots were incubated with secondary anti-mouse or anti-rabbit sera conjugated with horseradish peroxidase (Bio-Rad Laboratories, Hercules, CA) for 3 h at room temperature. The membranes were processed for protein detection using Super Signal™ substrate (Pierce, Rockford, IL,) to visualize proteins of interest by chemoluminescence. β-actin was used as a loading control, using a primary mouse antibody reactive to β-actin (#A-5441; Sigma-Aldrich). Protein concentrations were determined using the Bio-Rad protein assay kit according to the manufacturer's instructions.

### Tumor processing for histological sections

Tumors fixed in 10% buffered formalin were embedded in paraffin, using a tissue processor. Tumor samples were kept for 90 min in 70%, 95% and absolute ethanol, xylol and paraffin, and then remained in paraffin (70°C) until paraffin inclusion. The paraffin-embedded routine tissue blocks were stored at 4°C. Three and 4 µm-thick sections were cut from paraffin blocks onto superfrost ultra plus slides (Menzel-Glaser, Braunschweig, DE), for later use in Ki-67 immunohistochemistry and DNA fragmentation assay, respectively.

### *In situ* detection and quantitation of apoptosis

Apoptotic cells were quantitated in tumor tissue sections using the transferase mediated deoxyuridine triphosphate (dUTP)-digoxigenin nick-end labeling (TUNEL) assay [ApopTag® Red for indirect immunofluorescence staining kit (#S7165 Chemicon)], following the manufacturer's instructions. Specimens were then counterstained with Hoechst 5 µg/ml, for 10 min, at room temperature. Finally, slides were rinsed, dehydrated and a glass coverslip was mounted using Fluoromount-GTM mounting media (Beckman Coulter Inc., Fullerton, CA). The specimens were examined using a bright field microscope using a Axio Scope A.1 fluorescence microscope (Zeiss Axioskop; Carl Zeiss GmbH, Jena, Germany). Images were acquired, under a 100x magnification, using a DFC490 camera (Leica Microsystems AG, Heersbrugg, Switzerland) with the IM50 software for image acquisition (Leica Microsystems, version 1.20, Release 9). Positive apoptotic cells were detected, and their frequency compared within the different samples. Apoptosis index was evaluated in tumor section images at 100x, only being considered areas with dense tumor cell mass, displaying similar cell density between *Over-143* and *Empty* xenografts. Quantitation of TUNEL positive cells and tumor area were performed using Image J software (http://rsbweb.nih.gov/ij/). Apoptosis was expressed as number of TUNEL-positive cells per 10^3^ µm^2^ of tumor tissue.

### Immunohistochemistry for Ki-67 expression

Immunostaining was performed by the peroxidase-indirect-polymer method. Tumor tissue sections were deparaffinized, rehydrated and subjected to epitope antigen retrieval (20 min, 94°C) with target retrieval solution high pH 50x Dako Envision™ Flex (DAKO A/S, Glostrup, Denmark) in a pre-treatment module PTlink (Dako, Model PT 10130). Primary monoclonal mouse antibody anti-human KI67 (Clone MIB-1, #M7240; Dako) at 1∶300 was used. Immunohistochemistry was performed using an automated stainer (Dako Autostainer, Link 48) by the peroxidase-indirect-polymer method (#K8000, Dako) for Ki-67. Tonsil was used as positive control. For negative controls, the primary antibody was omitted during the staining. Proliferation was evaluated in tumor section images at 400x, only considering areas with dense tumor cell mass, displaying similar cell density between *Over-143* and *Empty* xenografts. Quantitation of Ki-67 positive cells was performed using Image J software. Proliferation was expressed as percentage of Ki-67-positive cells.

### Densitometry and statistical analysis

The relative intensifies of protein bands were analyzed using the densitometric analysis program Quantity One version 4.6 (Bio-Rad Laboratories). All data are expressed as the mean±SEM of similar samples, from two independent experiments. Statistical significance was evaluated using the Student’s t-test. Values of *p*<0.05 were considered statistically significant.
